# A systematic review and meta‐analysis of randomized controlled trials of endovascular thrombectomy compared with best medical treatment for acute ischemic stroke

**DOI:** 10.1111/ijs.12618

**Published:** 2015-08-26

**Authors:** Joyce S. Balami, Brad A. Sutherland, Laurel D. Edmunds, Iris Q. Grunwald, Ain A. Neuhaus, Gina Hadley, Hasneen Karbalai, Kneale A. Metcalf, Gabriele C. DeLuca, Alastair M. Buchan

**Affiliations:** ^1^ Centre for Evidence Based MedicineUniversity of OxfordOxfordUK; ^2^ Norfolk and Norwich University Teaching Hospital NHS TrustNorwichUK; ^3^ Acute Stroke ProgrammeRadcliffe Department of MedicineUniversity of OxfordOxfordUK; ^4^ NeuroscienceFaculty of Medical SciencePost Graduate Medical InstituteAnglia Ruskin UniversityChelmsfordUK; ^5^ Southend University Hospital NHS Foundation TrustSouthend‐on‐SeaUK; ^6^ CardioVascular Center Frankfurt (CVC Frankfurt)FrankfurtGermany; ^7^ Medical Sciences DivisionUniversity of OxfordOxfordUK; ^8^ Nuffield Department of Clinical NeurosciencesUniversity of OxfordOxfordUK; ^9^ Acute Vascular Imaging CentreUniversity of OxfordOxfordUK

**Keywords:** endovascular therapy/treatment, intravenous thrombolysis, ischemic stroke, meta‐analysis, systematic review, thrombectomy

## Abstract

**Background:**

Acute ischemic strokes involving occlusion of large vessels usually recanalize poorly following treatment with intravenous thrombolysis. Recent studies have shown higher recanalization and higher good outcome rates with endovascular therapy compared with best medical management alone. A systematic review and meta‐analysis investigating the benefits of all randomized controlled trials of endovascular thrombectomy where at least 25% of patients were treated with a thrombectomy device for the treatment of acute ischemic stroke compared with best medical treatment have yet to be performed.

**Aim:**

To perform a systematic review and a meta‐analysis evaluating the effectiveness of endovascular thrombectomy compared with best medical care for treatment of acute ischemic stroke.

**Summary of review:**

Our search identified 437 publications, from which eight studies (totaling 2423 patients) matched the inclusion criteria. Overall, endovascular thrombectomy was associated with improved functional outcomes (modified Rankin Scale 0–2) [odds ratio 1·56 (1·32–1·85), *P* < 0·00001]. There was a tendency toward decreased mortality [odds ratio 0·84 (0·67–1·05), *P* = 0·12], and symptomatic intracerebral hemorrhage was not increased [odds ratio 1·03 (0·71–1·49), *P* = 0·88] compared with best medical management alone. The odds ratio for a favorable functional outcome increased to 2·23 (1·77*–*2·81, *P* < 0·00001) when newer generation thrombectomy devices were used in greater than 50% of the cases in each trial.

**Conclusions:**

There is clear evidence for improvement in functional independence with endovascular thrombectomy compared with standard medical care, suggesting that endovascular thrombectomy should be considered the standard effective treatment alongside thombolysis in eligible patients.

## Introduction

Intravenous (IV) thrombolysis with recombinant tissue‐type plasminogen activator (rt‐PA) is currently the standard medical treatment for patients with acute ischemic stroke (AIS). However, recanalization rates with IV rt‐PA are usually low, particularly for major vessel occlusion [basilar artery, internal carotid artery, and middle cerebral artery 1, 2 ]. An earlier meta‐analysis (MA) with IV rt‐PA has demonstrated recanalization rates as low as 14% for internal carotid artery and 55% for middle cerebral artery occlusions 3. Similarly, a systematic review reported a recanalization rate of only 10–15% for major artery occlusions and a 20–40% success rate in a cohort of AIS patients treated with IV thrombolysis 4. While recanalization with IV rt‐PA is correlated with improved functional outcome, the low recanalization rates mean that a significant proportion of treated patients are not benefitting from this treatment.

The low rate of recanalization for large vessel occlusion instigated a search for improved recanalization strategies such as endovascular therapy (EVT). EVT can include rt‐PA delivered directly into the artery or retracting a clot using aspiration or a stent retriever. EVT is thought to produce higher recanalization rates, improve functional outcome and reduce mortality in patients with AIS. Despite higher recanalization rates, early randomized controlled trials (RCTs) of EVT failed to show any benefits of endovascular treatment over IV thrombolysis alone 5, 6, 7. The use of older generation devices, lack of documented vessel occlusion before treatment, and time delay were some of the factors responsible for the failure of these EVT trials. However, recent clinical data from multiple RCTs that evaluated endovascular thrombectomy over best medical treatment (which may or may not include IV rt‐PA) for patients with major vessel occlusion in AIS have shown overwhelming evidence in favor of mechanical thrombectomy. The first convincing evidence of the benefit of endovascular thrombectomy for the management of stroke came from the Multicenter Randomized Clinical Trial of Endovascular Treatment for Acute Ischemic Stroke in the Netherlands (MR CLEAN) 8. This was subsequently supported by other trials: the Endovascular Treatment for Small Core and Anterior Circulation Proximal Occlusion With Emphasis on Minimizing CT to Recanalization Times (ESCAPE) 9, Extending the Time for Thrombolysis in Emergency Neurological Deficits – Intra‐Arterial (EXTEND‐IA) 10, Randomized Trial of Revascularization With the Solitaire FR Device Versus Best Medical Therapy in the Treatment of Acute Stroke Due to Anterior Circulation Large Vessel Occlusion (REVASCAT) 11, and Solitaire With the Intention for Thrombectomy as Primary Endovascular Treatment Trial (SWIFT PRIME) 12 studies.

The findings in these recent trials suggest a superior outcome following treatment with IV thrombolysis and thrombectomy using modern thrombectomy devices compared with best medical treatment alone. The successes from the new RCTs have been attributed mostly to improved thrombectomy devices with faster and higher rates of recanalization and better study protocols with documentation of vessel occlusion before randomization. This is in addition to improved time to revascularization.

While previous systematic reviews (SRs) and MA failed to show any superiority or benefit of EVT over IV thrombolysis for AIS 13, 14, 15, a recent SR and MA involving all the previous prospective studies, including MR CLEAN (but excluding ESCAPE, EXTEND‐IA, REVASCAT, and SWIFT PRIME), demonstrated superiority of EVT over best medical treatment alone for AIS 16. The recent publication of these additional four RCTs has prompted the need for an updated SR and MA to amalgamate the latest evidence comparing EVT using mechanical thrombectomy devices over medical treatment alone. The aim of this study is to perform a comprehensive SR and MA evaluating RCTs of AIS management comparing thrombolysis to EVT wherein at least 25% of EVT cases were treated with thrombectomy devices.

## Methods

This study was guided by established review protocols of the Cochrane Collaboration 17 and followed the PRISMA standard 18 to report findings.

### Search strategy and selection criteria

Studies were identified via electronic searches in databases (Medline, EMBASE, Cochrane Database of Systematic Reviews, and the Cochrane Central Registry of Controlled Trials), cross‐referencing and hand‐searching reference lists of relevant journals, and were published between January 1995 and May 2015. The search terms used were ‘brain isch(a)emia’, ‘acute isch(a)emic stroke’, ‘cerebral infarction’, ‘cerebrovascular accident’, ‘CVA’, AND ‘mechanical thrombolysis’, ‘endovascular therapy’, ‘endovascular treatment’, ‘endovascular embolectomy’, ‘endovascular thrombectomy’, ‘intra(‐)arterial intervention’, ‘intra(‐)arterial treatment’, ‘intra(‐)arterial therapy’, ‘intra(‐)arterial thrombolysis’, ‘neuro(‐)thrombectomy’, AND ‘randomized controlled trial’ (where parentheses indicate terms that were searched with and without the bracketed text). Studies of any language were considered.

### Eligibility criteria

Studies were included if they were RCTs meeting the following criteria:

- Completed studies were published in peer‐reviewed journals,
- Study populations were greater than 20 cases,
- Patients had AIS due to major vessel occlusion and had received treatment with endovascular intervention, IV thrombolysis or best medical care, or endovascular treatment with IV thrombolysis,
- Major vessel occlusion was confirmed by CT angiography or MR angiography,
- In studies with endovascular thrombectomy, both old (MERCI) and new generation (Stent retrievers and Penumbra aspiration 5 MAX, ACE) devices were utilized in at least 25% of cases (pure manipulation of the clot with a guide wire, without use of a thrombectomy device, was not considered endovascular thrombectomy),
- The studies reported the following outcomes: functional outcome [measured by the modified Rankin Scale (mRS)], all‐cause mortality, and symptomatic intracerebral hemorrhage (sICH), and
- The studies reported a risk estimate [relative risk or odds ratio (OR)] or had available data for the calculation of a risk estimate.

### Study selection and analysis

**Figure 1:**
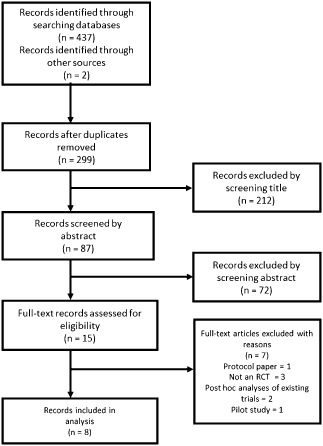
PRISMA flow chart showing study selection.

The titles and abstracts of studies were examined by four reviewers (J. S. B., B. A. S., G. H., A. A. N.), and then full texts were scrutinized if necessary for inclusion. Eligibility assessment and data extraction were performed independently by five reviewers (J. S. B., B. A. S., L. D. E., A. A. N., G. H.) and entered into a standard format. Researchers then met to discuss areas of agreement and completeness, and disagreements were resolved by consensus. Figure 1 shows the PRISMA flow chart of study selection. Study quality was assessed using the CASP Randomized Controlled Trial checklist 19.

### Data extraction

The following information was extracted from the included primary studies as well as published supplementary materials:

- background characteristics of the included studies (trial and first author, trial period, location, number of patients, number of centers, and devices used) (Table 1),
- main characteristics of patients [age and gender, stroke severity on admission as measured with the National Institutes of Health Stroke Scale (NIHSS)], time from symptom onset to treatment, recanalization as measured by TICI Score (2b/3), primary outcome (mRS 0–2 at 90 days), and secondary outcomes (mortality and sICH) (Table 2),
- secondary characteristics [onset to groin puncture, CT to groin puncture, onset to randomization, baseline Alberta Stroke Program Early CT Score (ASPECTS), serious adverse effects, and NIHSS score post treatment] (Table 3),
- proportion of EVT patients that underwent mechanical thrombectomy using a thrombectomy device (Table 4), and
- outcome measure data as described below.

**Table 1:** Background characteristics of the included studies

Trial, first author, year (reference)	Trial period	Location	Total no. of patients	No. of centers	Device
ESCAPE Goyal, 2015 9	2013*–*2014	North America, Europe, South Korea	315	22	Stent retrievers, Solitaire FR Device
EXTEND 1A Campbell, 2015 10	2012*–*2014	Australia and New Zealand	70	14	Solitaire FR Device
IMS III Broderick, 2013 5	2006–2012	North America, Europe, Australia	656	58	Merci Retriever, Penumbra System, Solitaire FR Device, Micro Sonic
MR CLEAN Berkhemer, 2015 8	2010–2014	Europe	500	30	Retrievable stents, other devices (not specified)
MR RESCUE Kidwell, 2013 7	2004–2011	North America	118	22	Merci Retriever, Penumbra System
REVASCAT Jovin, 2015 11	2012*–*2014	Spain	206	4	Solitaire FR Device
SWIFT PRIME Saver, 2015 12	2013–2014	North America, Europe	196	39	Stent retrievers
SYNTHESIS Ciccone, 2013 6	2008–2012	Europe	362	24	Solitaire FR Device, Penumbra System, Trevo device, Merci Retriever

**Table 2:** Summary of included studies: main characteristics

Trial, year	Age (y) (mean ±) or median (IQ range)	No. patients (male %)	Admission NIHSS score, mean/median (range)	Occlusion sites (ICA, ICA + M1, M1, M1 + M2, M2, A1, or A2)	Time from symptom onset to treatment (min), median/mean ± SD	Recanalization (TICI score) 2b/3	Primary outcome (mRS 0–2) at 90 days or mean (CI)	Secondary outcomes Mortality, sICH
ESCAPE, 2015 9								
IV	70 (60–81)	150 (47·3)	17 (12*–*20)	ICA + M1 = 39, M1 + M2 = 105, M2 = 3	N/A	31·2% (AOL score)	29·3%	19·0%, 2·7%
EVT	71 (60–81)	165 (47·9)	16 (13*–*20)	ICA + M1 = 45, M1 +, M2 = 111, M2 = 6	200	72·4% (TICI 2b/3)	53·0%	10·4%, 3·6%
EXTEND IA, 2015 10								
IV	70·2 ± 11·8	35 (49)	13 (9–19)	ICA = 11, M1 = 18, M2 = 6	N/A		40·0%	20%, 6·0%
EVT	68·6 ± 12·3	35 (49)	17 (13*–*20)	ICA = 11, M1 = 20, M2 = 4	210 (166*–*251)^†^	86·0% (25/29 pts)	71·0%	9·0%, 0%
IMS III, 2013 5								
IV	68 (23*–*84)	222 (55·0)	16 (8–30)	LH: 106 (47·7); RH: 109 (49·1); BS: 4 (1·8); UnK: 3 (1·4)	122·4 ± 33·7		40·2%	21·6%, 5·9%
EVT	69 (23*–*89)*	434 (50·2)	17 (7–40)	LH: 224 (51·6); RH: 197 (45·4); BS: 10 (2·3); UnK: 3 (0·7)	249·4 ± 50·6	38·0% (ICA), 44·0% (M1), 44·0% (single M2), 23·0% (multiple M2)	42·7%	19·1%, 6·2%
MR CLEAN, 2015 8								
IV	65·7 (55·5–76·4)	267 (58·8)	18 (14*–*22)	ICA = 3, ICA + M1 = 75, M1 = 165, M2 = 21, A1 or A2 = 2; ExCr = 70	N/A		19·1%	22%, 6·4%
EVT	65·8 (54·5–76·0)	233 (57·9)	17 (14*–*21)	ICA = 1, ICA + M1 = 59, M1 = 154, M2 = 18, A1 or A2 = 1; ExCr 75	260 (210–313)^†^	58·7%	32·6%	21%, 7·7%
MR RESCUE, 2013 7								
IV								
Penumbral	65·8 ± 16·9	34 (44)	16 (11–18)	ICA = 5, M1 = 23, M2 = 6	348 ± 60^‡^	52·0%	26·0%	21·0%, 6%
Non‐penumbral	69·4 ± 15·9	20 (60)	20·5 (17–23)	ICA = 2, M1 = 16, M2 = 2	342 ± 84^‡^	20·0%	10·0%	30·0%, 0%
EVT								
Penumbral	66·4 ± 13·2	34 (50)	16 (12–18)	ICA = 6, M1 = 18, M2 = 10	318 ± 96^‡^	57·0%	21·0%	18·0%, 9%
Non‐penumbral	61·6 ± 12·0	30 (43)	19 (17–22)	ICA = 7, M1 = 21, M2 = 2	312 ± 84^‡^	37·0%	17·0%	20·0%, 0%
REVASCAT, 2015 11								
IV	67·2 ± 9·5	103 (52·4)	17·0 (12–19)	ICA = 1, ICA + M1 = 27, M1 = 65, M2 = 8			28·0%	16·0%, 1·9%
EVT	65·7 ± 11·3	103 (53·4)	17·0 (14–20)	ICA + M1 = 26, M1 = 66, M2 = 10	269 (201–340)^†^	66·0%	44·0%	18·0%, 1·9%
SWIFT PRIME, 2015 12								
IV	66·3 ± 11·3	98 (47)	17 (13*–*19)	ICA = 15, M1 = 76, M2 = 62			36·0%	12·0%, 3·0%
EVT	65·0 ± 12·5	98 (55)	17 (13*–*20)	ICA = 17, M1 = 6, M2 = 13	224^†^	88·0%	60·0%	9·0%, 0%
SYNTHESIS, 2013 6								
IV	67 ± 11	181 (57)	13 (9–18)	N/A	165 (140*–*200)		46·4%	6·0%,6·0%
EVT	66 ± 11	181 (59)	13 (9–17)	N/A	225 (194*–*440)	N/A	42·0%	8·0%,6·0%

Key IV, intervention group; EVT, thrombectomy group. *Complete age range reported. ^†^Onset to groin puncture. ^‡^Time to enrolment.

**Table 3:** Summary of included studies: secondary characteristics

Trial, year published	Onset to groin puncture (min)	CT to groin puncture (min)	Onset to randomization (min)	ASPECT median (interquartile range)	NIHSS score, mean/median Post treatment
ESCAPE, 2015 9					24 h:
IV			172 (119*–*284)	9 (8–10)	13 (6–18)
EVT	200	51 (39*–*68)	169 (117*–*285)	9 (8–10)	6 (3–14)
EXTEND 1A, 2015 10					Only given as reduction from baseline
IV					
EVT	210 (166*–*251)	93 (71–138)	Not given	Not given	
IMS III, 2013 5					Used for stratification for mRS; full data not presented
IV					
EVT	208 (SD 46·7)	N/A	N/A	Not given	
MR CLEAN, 2015 8					24 h:
IV			196 (149*–*266)	9 (8–10)	16 (12*–*21)
EVT	260 (210*–*313)	Not given	204 (152*–*251)	9 (7–10)	13 (6–20)
MR RESCUE, 2013 7					
IV					
Penumbral					
Non‐penumbral					
EVT	330	N/A	N/A	Not given	Not given
Penumbral					
Non‐penumbral					
REVASCAT, 2015 11					At 90 days:
IV			226 (168–308)	8 (6–9)	6·0 (2·0–11·0)
EVT	269 (201–340)	N/A	223 (170–312)	7 (6–9)	2·0 (0·0–8·0)
SWIFT PRIME, 2015 12					At 27 h:
IV				9 (8–10)	−8·5 ± 7·1
EVT	224	58 (41*–*83)	N/A	9 (7–10)	−3·9 ± 6·2
SYNTHESIS, 2013 6					Day 7 (or at discharge):
IV	N/A	N/A	145 (119–179)		13 (9–18)
EVT	N/A	N/A	148 (124–190)	Not given	13 (9–17)

**Table 4:** Number of patients treated with a thrombectomy device in the endovascular thrombectomy group

Trial	No. of patients in endovascular treatment group	No. of patients with thrombectomy device	% of Patients with thrombectomy device
ESCAPE	165	130	79
EXTEND IA	35	27	77
IMS III	434	170	39
MR CLEAN	233	190	82
MR RESCUE	64	61	95
REVASCAT	103	98	95
SWIFT PRIME	98	87	89
SYNTHESIS	181	56	31

### Outcome measures

The prespecified primary outcome measure was clinical functional independence as determined by an mRS score of 0–2 at 90 days. The prespecified secondary outcomes were sICH as defined by the trials and all‐cause mortality at 90 days. Other outcomes of interest included: the number of patients who were able to walk unassisted but with increasing levels of disability from none to moderate (mRS 0*–*3) at 90 days; and the number of patients who had mRS 0*–*2 at 90 days with a baseline ASPECTS 8–10 (minimal evidence of underlying ischemic change), ASPECTS 5*–*7 (moderate evidence of underlying ischemic change), or a baseline ASPECTS 0*–*4 (substantial evidence of underlying ischemic change).

### Data analyses

All MAs were conducted using Review Manager version 5.3 (The Nordic Cochrane Centre, The Cochrane Collaboration, Copenhagen, Denmark). MAs were performed for the prespecified primary and secondary outcomes with the results stratified by the administered treatment type. The ORs and 95% confidence intervals were calculated from the extracted data using the Mantel–Haenszel fixed‐effects model. The heterogeneity between studies was tested using the inconsistency index (*I* ^2^): values less than 25% were considered low; between 25 and 70% were considered moderate; and greater than 70% were considered highly heterogeneous 20. A DerSimonian and Laird's random effect model was only used where there was a high level of heterogeneity between studies. A *P* value of <0·05 was considered statistically significant.

## Results

### Study characteristics

The search yielded 437 studies, eight of which were retained for MA (Fig. 1). The eight RCTs comparing endovascular therapies with IV thrombolysis meeting the eligibility criteria included ESCAPE 9, EXTEND‐IA 10, IMS III 5, MR CLEAN 8, MR RESCUE 7, REVASCAT 11, SWIFT PRIME 12, and SYNTHESIS 6. THERAPY 21 and THRACE 22, trials still awaiting publication, were not incorporated in this analysis. 1 Seven of the studies were multicenter trials based in several countries with REVASCAT the only trial to be conducted in one country (Spain). Patient populations ranged from *n* = 70 to *n* = 656, with a total of 2423 patients; 1313 patients were randomized to EVT, with mean age ranging from 65 to 71 years, and 1110 patients to IV thrombolysis with mean age ranging from 66 to 70 years. The median baseline NIHSS ranged from 13 to 21 for the IV thrombolysis group and 13–19 for the EVT group. Different thrombectomy devices were used in the RCTs, with newer generation devices being used in more recent trials (Table 1). The characteristics of the selected studies are summarized in Tables 1 –4.

### MA of outcome measures

Two sets of MAs were conducted. The first analysis was performed on the eight identified studies (Table 1). The second analysis was conducted on the six published studies that had >50% of patients in the EVT group treated by mechanical thrombectomy with MERCI and new generation devices (ESCAPE, EXTEND, MR CLEAN, MR RESCUE, REVASCAT, and SWIFT PRIME) (Table 4).

### Primary outcome

**Figure 2:**
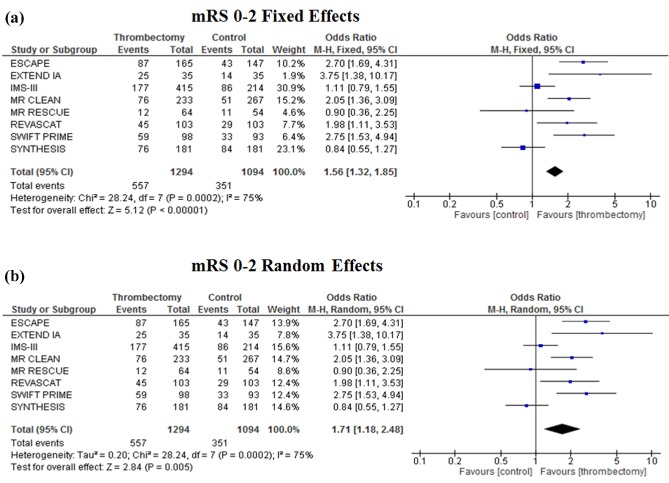
Meta‐analysis of primary outcome (mRS 0*–*2) of patients treated with endovascular thrombectomy compared with intravenous thrombolysis for acute ischemic stroke using a fixed effect model (a) and a random effect model (b).

Patients treated with EVT had a significantly greater chance of a favorable prespecified primary outcome (mRS 0–2 at 90 days) [OR 1·56 (95% CI, 1·32–1·85), *P* < 0·00001] than those treated with best medical treatment alone (Fig. 2a). However, there was moderate heterogeneity among studies (*I* ^2^ = 75%, *P* = 0·0002), and so, a random effect analysis was performed (Fig. 2b), which still showed improved functional independence [OR 1·71 (1·18*–*2·48), *P* = 0·005] in favor of EVT. There was no evidence of publication bias based on the symmetrical funnel plot (Fig. S1) for the primary outcome (mRS 0–2 at 90 days).

### Secondary outcome

**Figure 3:**
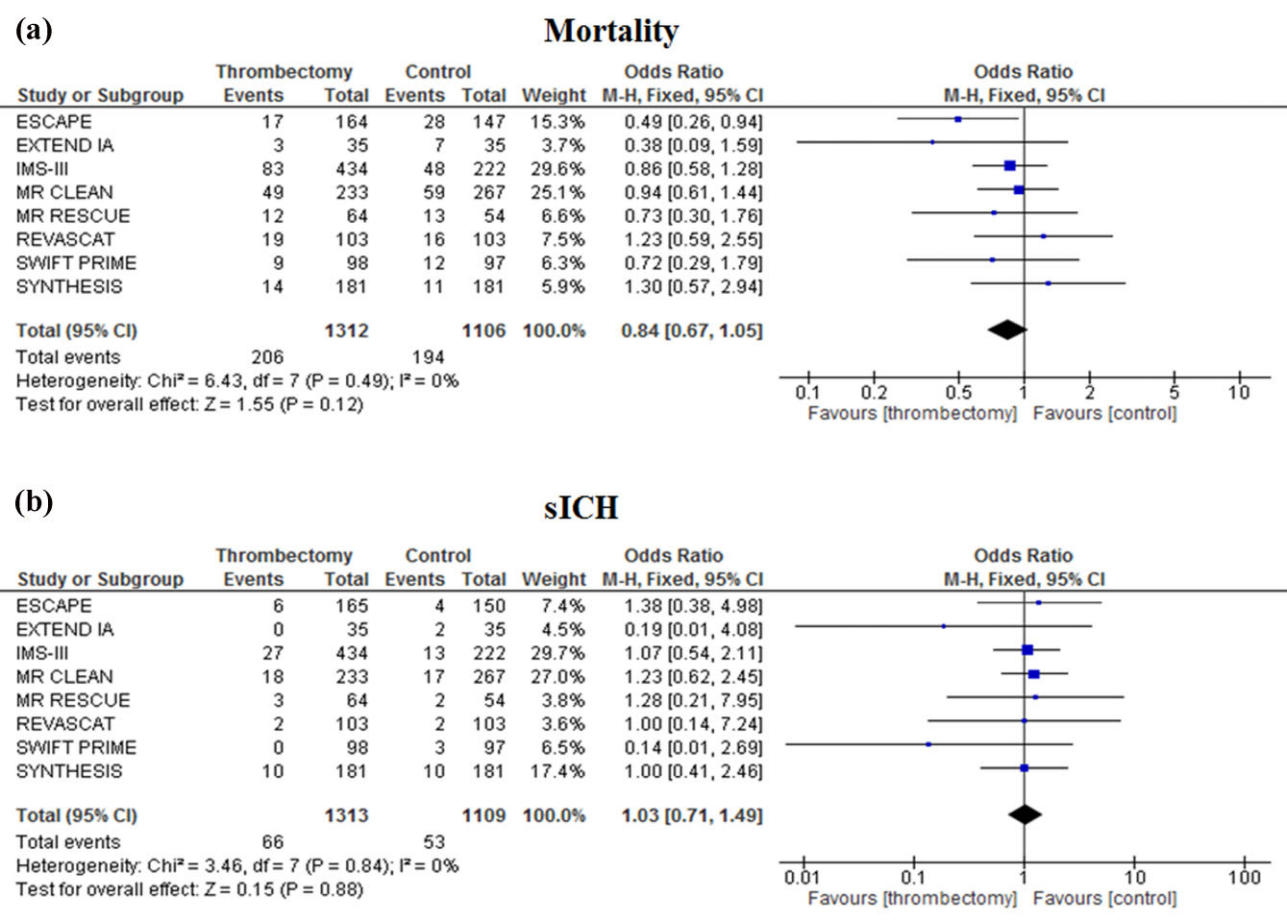
Meta‐analysis of secondary outcome measures, mortality at 90 days (a) and symptomatic intracerebral hemorrhage (sICH) (b), of patients treated with endovascular thrombectomy compared with intravenous thrombolysis for acute ischemic stroke.

No significant difference was found in the prespecified secondary outcomes mortality and sICH when EVT was compared with IV rt‐PA. EVT had a tendency toward decreased mortality at 90 days [OR 0·84 (0·67–1·05), *P* = 0·12] compared with best medical management alone (Fig. 3a). EVT did not increase the incidence of sICH [OR 1·03 (0·71–1·49), *P* = 0·88; Fig. 3b]. Unlike theprimary outcome, no heterogeneity was noted between studies for the secondary outcomes (*I* ^2^ = 0% for both mortality and sICH).

### Other analyses

#### mRS 0*–*3 at 90 days

**Figure 4:**
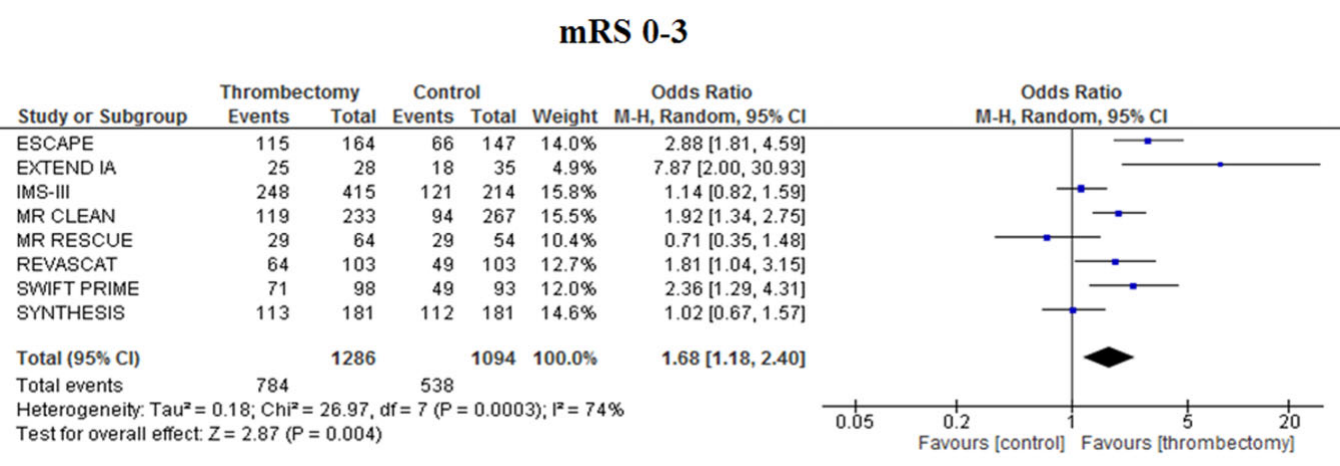
Meta‐analysis of mRS 0*–*3 in patients treated with endovascular thrombectomy compared with intravenous thrombolysis for acute ischemic stroke.

EVT improved functional independence (mRS 0–3) at 90 days compared with best medical management alone [OR 1·68 (1·18–2·40), *P* = 0·004] with significant heterogeneity between studies (*I* ^2^ = 74%, *P* = 0·0003) (Fig. 4).

#### Subgroup analysis of RCTs with >50% of EVT patients treated with a thrombectomy device

**Figure 5:**
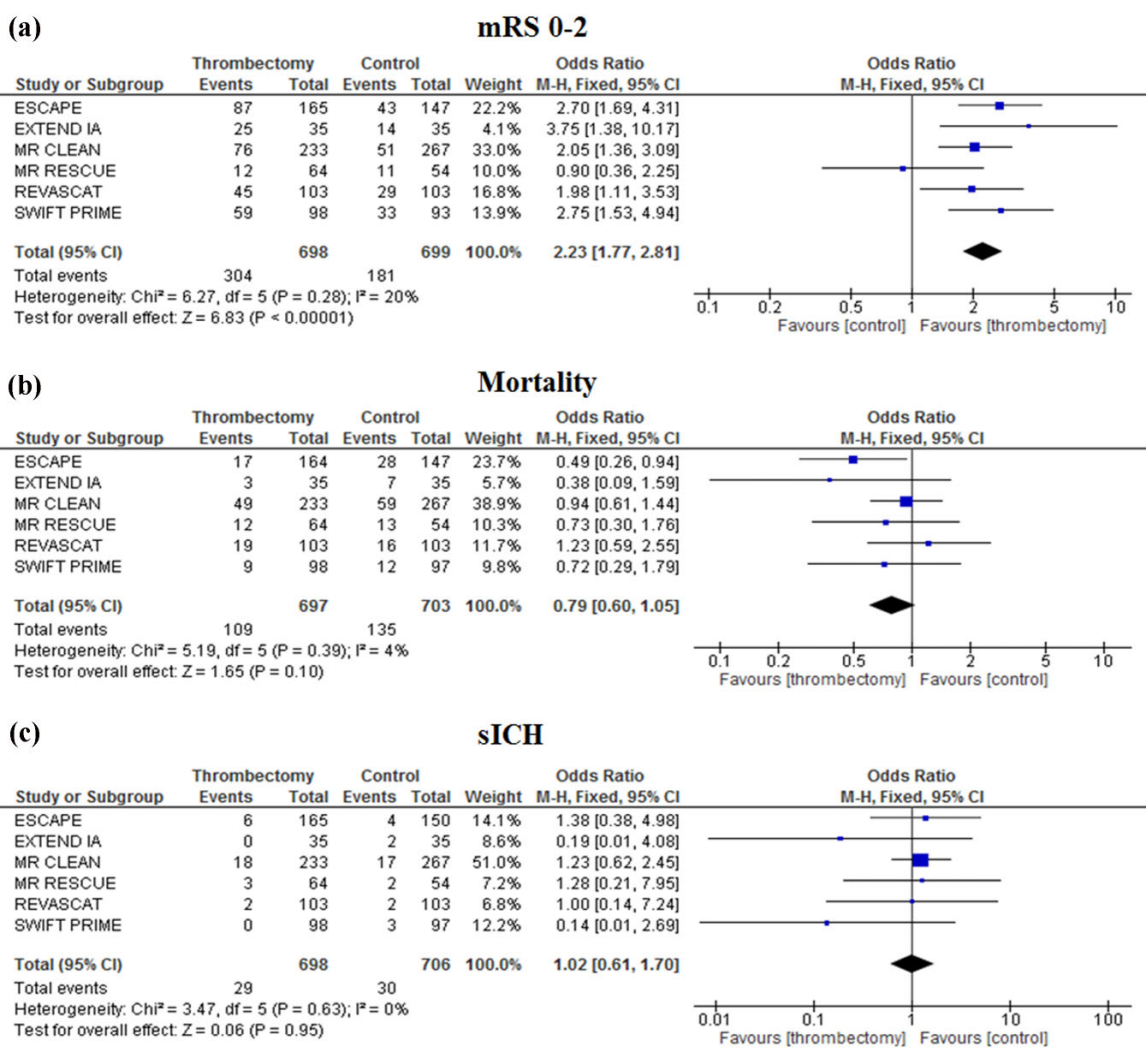
Subgroup meta‐analysis of trials with greater than 50% thrombectomy in the endovascular treatment group. Outcome measures analyzed include mRS 0*–*2 at 90 days (a), mortality at 90 days (b), and symptomatic intracerebral hemorrhage (c).

In the sub‐group analysis involving the six RCTs with >50% of endovascular patients treated with a thrombectomy device, there was an increase in the OR [2·23 (1·77*–*2·81), *P* < 0·00001] for a favorable outcome in the EVT group compared with best medical management alone (Fig. 5a). Treatment with mechanical thrombectomy did not significantly reduce 90‐day mortality [OR 0·79 (0·60–1·05), *P* = 0·10; Fig. 5b]. Similarly, EVT did not increase sICH compared with best medical treatment alone [OR 1·02 (0·61–1·70), *P* = 0·95; Fig. 5c]. No heterogeneity was noted between studies for these outcomes (*I* ^2^ = 20% for mRS score 0–2, 4% for mortality, and 0% for sICH).

### Subgroup analysis with ASPECTS

**Figure 6:**
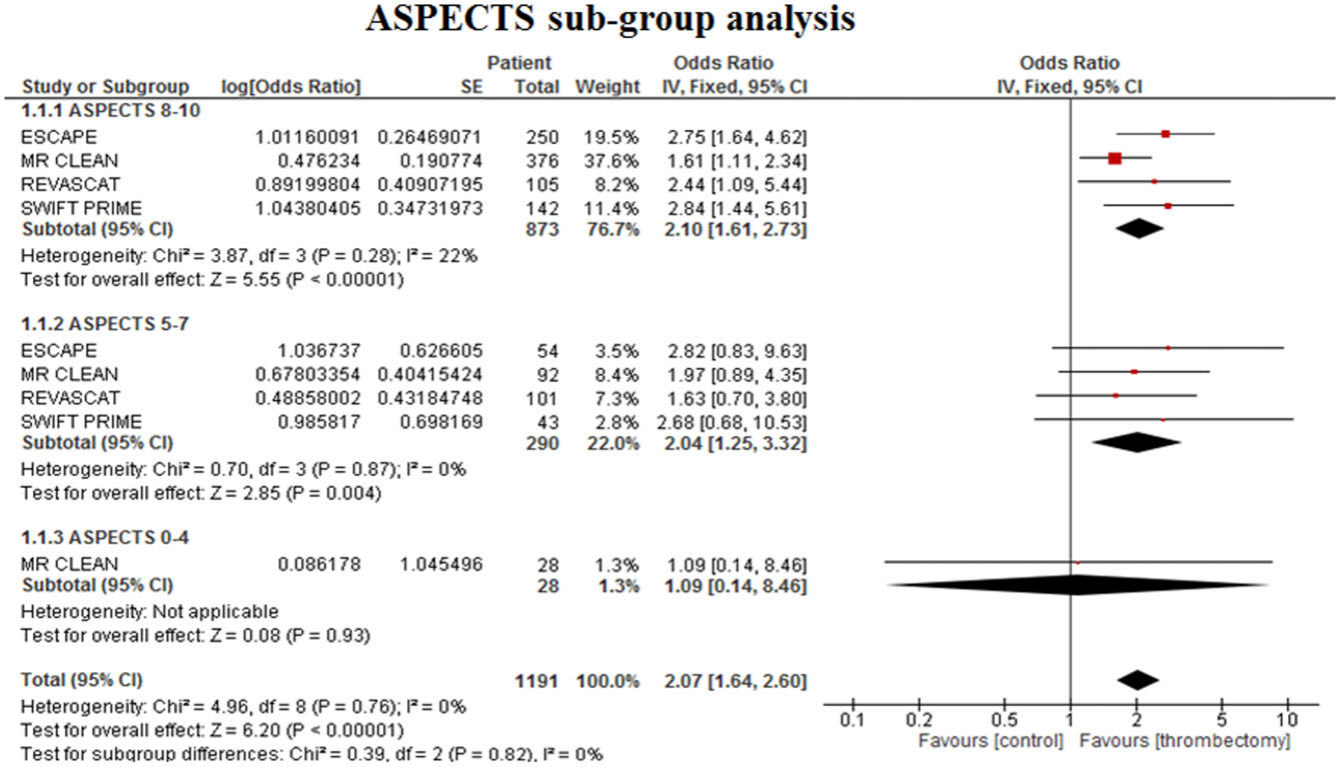
Subgroup meta‐analysis of functional outcome (mRS 0*–*2) at 90 days for baseline ASPECTS 8–10 (minimal evidence of underlying ischemic change), ASPECTS 5*–*7 (moderate evidence of underlying ischemic change), and baseline ASPECTS 0*–*4 (substantial evidence of underlying ischemic change).

This subgroup analysis divided each treatment arm into three subgroups: baseline ASPECTS 8–10 (minimal evidence of underlying ischemic change), baseline ASPECTS 5–7 (moderate evidence of underlying ischemic change), and baseline ASPECTS 0–4 (substantial evidence of underlying ischemic change) 23. Four studies were included in this analysis as these were the only studies that presented stratified ASPECTS data for mRS [ESCAPE 9, MR CLEAN 8, REVASCAT 11, and SWIFT PRIME 12 ]. IMS III 5 presented stratified data for ASPECTS 8–10 and 0–7, but due to the difference in stratification and having <50% of EVT patients undergoing thrombectomy, this study was excluded from this subgroup MA. EVT improved functional independence compared with best medical treatment in patients with high baseline ASPECTS [OR 2·10 (1·61–2·73), *P* < 0·00001; Fig. 6 ]. EVT also improved functional independence with moderate baseline ASPECTS [OR 2·04 (1·25–3·32), *P* = 0·004]. There was no evidence of benefit of EVT in patients with low baseline ASPECTS [OR 1·09 (0·14–8·46), *P* = 0·93], but only 28 patients were included in this analysis due to MR CLEAN being the only study that incorporated this group of patients in their trial. Overall, these results suggest that patients with baseline ASPECTS >4 benefit from EVT.

## Discussion

These SR and MA combine the results from all published RCTs comparing EVT (where at least 25% of patients were treated with a thrombectomy device) to best medical management alone for the treatment of AIS. Eight trials (2423 patients) of EVT compared with best medical treatment alone were identified and included in this analysis. In the combined MA of the eight trials, EVT demonstrated improved clinical functional outcome (number of patients with mRS 0–2 at 90 days poststroke), but there was high heterogeneity between the studies. The heterogeneity could be accounted for by different study designs, times to treatment and differing imaging methodologies, as well as the use of newer generation thrombectomy devices with higher recanalization rates in the more recent trials. There was a tendency toward decreased mortality with EVT compared with best medical treatment alone, indicating the benefit of this therapeutic strategy. In addition, sICH incidence was not increased with EVT suggesting that the use of thrombectomy devices alongside IV rt‐PA does not increase the risk of hemorrhage compared with IV rt‐PA alone.

The eight primary trials included in the MA contained EVT groups where patients underwent either intra‐arterial rt‐PA therapy and/or mechanical manipulation of the clot (often not involving a thrombectomy device but wire manipulation or ultrasound without clot extraction). While earlier trials [SYNTHESIS 6 and IMS III 5 ] predominantly used intra‐arterial rt‐PA for EVT, the remaining six trials used a thrombectomy device in >50% of cases in the EVT group (Table 4). Therefore, we analyzed these trials with more frequent use of mechanical thrombectomy devices in a separate MA. The results showed that in these trials, the chances of a better outcome were superior to the primary MA (where only at least 25% of EVT patients needed to be treated with thrombectomy). There was also greater homogeneity between studies, which could be attributed to similar study design between these RCTs. Similar to the primary analysis, there was no difference in mortality or sICH rate between EVT and IV thrombolysis in the trials with substantial use of endovascular thrombectomy.

Five trials [ESCAPE 9, IMS III 5, MR CLEAN 8, REVASCAT 11, and SWIFT PRIME 12 ] reported baseline ASPECTS, a score that divides the brain into 10 regions and assesses each brain region for ischemic changes based on a CT image 23. A higher ASPECTS indicates minimal ischemic change, while a lower ASPECTS signifies substantial ischemic change. Subgroup analysis was only possible using data from ESCAPE 9, MR CLEAN 8, REVASCAT 11, and SWIFT PRIME 12 as these were the only studies that reported mRS scores stratified by high ASPECTS 8, 9, 10, moderate ASPECTS 5, 6, 7, or low ASPECTS (0*–*4). In fact, ESCAPE, REVASCAT, and SWIFT PRIME had baseline ASPECTS <6 as an exclusion criterion for their trials, and so the majority of ASPECTS 5–7 patients included in the MA would have been patients with scores of 6 or 7. The ASPECTS sub‐group MA showed that with both high and moderate baseline ASPECTS, the odds of mRS 0–2 at 90 days improved with EVT compared to IV rt‐PA, while this was not the case in patients with low (<5) baseline ASPECTS. The low baseline ASPECTS analysis only included 28 patients from MR CLEAN, and further analysis will be undertaken once more data have been published that includes patients stratified by ASPECTS. Until these data are available, we cannot confirm whether EVT is beneficial or not in this patient group.

The key aspect of EVT is the restoration of cerebral blood flow. In clinical trials, this is usually measured through the thrombolysis in cerebral infarction (TICI) scale, which assesses recanalization by digital subtraction angiography. In the majority of the eight trials identified, the recanalization score was reported, but unfortunately, MA was not possible due to variations in the comparisons made (EVT vs. IV thrombolysis or EVT vs. baseline) and the timing of measurements. These variations in the reporting of data from the TICI scale are evident throughout a number of other revascularization studies, making the interpretation and comparison of these scores across studies difficult 24. Time‐to‐recanalization could also be a critical contributor to outcome, where it was noted that MR RESCUE had the longest time‐to‐recanalization (>5 h) and did not show benefit, whereas the more recent trials with positive effects had a time‐to‐recanalization within three‐ to five‐hours. In addition to assessment of recanalization, some studies also examined the extent to which the brain reperfused following recanalization. In EXTEND‐IA 10 and SWIFT PRIME 12, EVT showed that reperfusion at 24 h poststroke (assessed by CT and MR perfusion imaging) was substantially improved and associated with better functional outcome compared with IV rt‐PA alone.

The secondary outcomes analyzed as part of the MA showed that EVT was safe (EVT does not increase sICH beyond that which is already produced by rt‐PA, and does not increase mortality) while producing a clear beneficial effect on clinical outcome (mRS score). The lack of adverse effects of endovascular thrombectomy highlights the applicability of this procedure as a therapeutic strategy for AIS.

There are a number of limitations of this MA. Like any MA, data were pooled from trials with differences in design and methodology, particularly with regard to patient selection, time to treatment, and the use of different mechanical devices which could all be sources of bias. Patients in the endovascular arm of the studies evaluated mainly received IV thrombolysis followed by mechanical clot extraction with or without the additional use of thrombolytics. There were also patients that did not undergo IV thrombolysis and received direct mechanical thrombectomy [e.g. 10% of patients in the MR CLEAN study 8 ]. As a result, the MA population was not perfectly homogeneous which was only evident when assessing all eight trials for the primary outcome. In addition, our inclusion criteria required that at least 25% of patients in the EVT arm were treated by mechanical thrombectomy with a device. Most studies met this inclusion criterion, but IMS III 5 and SYNTHESIS 6 had a much greater proportion of patients treated endovascularly by intra‐arterial rt‐PA or wire manipulation alone than mechanical thrombectomy. IMS III had several issues regarding the trial design and execution with lack of evidence of vessel occlusion, the use of older devices, and resultant lower recanalization rates. Despite the limitations of IMS III, it is repeatedly quoted as a comparator and also used in MA. As the time course of restoration of cerebral blood flow is disparate between rt‐PA (gradual) and thrombectomy (instant), these differences in endovascular treatment could have an effect on outcome. Also, with wire manipulation alone, vessel re‐opening rates are low, and no clot is extracted. Hence, our sub‐group analysis of trials that used mechanical thrombectomy in greater than 50% of patients in the EVT group showed a stronger positive effect on clinical outcome, with greater homogeneity between studies. A patient‐level MA would provide greater scope for further analysis, particularly when assessing contributing factors to outcome, but with these included studies, this was not possible.

Despite these limitations, this MA was able to evaluate the positive impact of mechanical thrombectomy on clinical outcome in patients with AIS secondary to major vessel occlusion. Given the rigor associated with angiographic and clinical assessments in all eight trials, this allowed data pooling for a number of primary and secondary outcomes and some sub‐group analyses where consistencies between studies were reached.

## Conclusions

Our study has shown overwhelming evidence of improved functional outcome in favor of EVT over medical treatment alone. EVT did not increase the incidence of sICH and showed marginal evidence for reduced mortality at 90 days, confirming the safety of the procedure. The benefit of EVT on functional independence was even more marked in the subgroup analysis of RCT trials where >50% of EVT patients underwent mechanical thrombectomy. Overall, the substantial evidence of improvement in functional outcome without an increased incidence of adverse effects means that EVT should now be considered as a primary treatment option for eligible AIS patients, alongside IV thrombolysis.

## Contributors

J. S. B. searched the literature, extracted the data, reviewed available studies, guided the review process, and wrote the paper. B. A. S. reviewed available studies, extracted the data, analyzed the results, and co‐wrote the paper. L. D. E. guided the literature search and data extraction process, searched the literature, extracted the data, contributed to and reviewed the paper. I. Q. G. extracted data and co‐wrote the paper. A. A. N. reviewed available studies, extracted the data, and reviewed the paper. G. H. reviewed available studies, extracted the data, and reviewed the paper. H. K. reviewed the paper. K. A. M. reviewed the paper. G. C. D. reviewed the paper. A. M. B. reviewed and made critical revisions of the paper.

## Supporting information


**Figure S1.** Funnel plot of included studies for the primary outcome (mRS 0–2) showing symmetry of studies suggestive of lack of publication bias.
**Table S1.** The complete search terms with Boolean operators included.
**Table S2.** All 299 studies showing reasons for exclusion.
**Table S3.** Quality of RCTs using the CASP Randomized Controlled Trials Checklist.

## Footnote

1 The authors are aware of THERAPY and THRACE, randomized controlled trials on EVT for AIS that are undergoing final analysis before publication. The trials’ results were presented at the European Stroke Organisation Conference (Glasgow, United Kingdom, April 17, 2015), and while the trials meet the eligibility criteria for MA, the results were not included here.
