# Supplementary material for: A systematic review and meta‐analysis of randomized controlled trials of endovascular thrombectomy compared with best medical treatment for acute ischemic stroke
Source: Int J Stroke. 2015 Aug 26;10(8):1168–78. doi: 10.1111/ijs.12618 (PMC5102634; doi:10.1111/ijs.12618)
Supplement: Supplementary file 1 — Figure S1. Funnel plot of included studies for the primary outcome (mRS 0–2) showing symmetry of studies suggestive of lack of publication bias. Table S1. The complete search terms with Boolean operators included. Table S2. All 299 studies showing reasons for exclusion. Table S3. Quality of RCTs using the CASP Randomized Controlled Trials Checklist. [file IJS-10-1168-s001.docx]

**Supplementary Information**

Table S1: the complete search terms with Boolean operators included

| **Incident** | **Treatment** | **Study design** |
| --- | --- | --- |
| Brain ischaemia OR  brain ischemia OR  acute ischaemic stroke OR acute ischemic stroke OR Cerebral infarction OR cerebrovascular accident OR CVA  AND | Mechanical thrombolysis OR endovascular therapy OR endovascular treatment OR endovascular embolectomy OR endovascular thrombectomy OR  intra-arterial intervention OR intraarterial intervention OR intra-arterial treatment OR intraarterial treatment OR intra-arterial therapy OR intraarterial therapy OR  intra-arterial thrombolysis OR intraarterial thrombolysis OR neurothrombectomy OR neuro-thrombectomy  AND | Randomized Controlled trial |

**
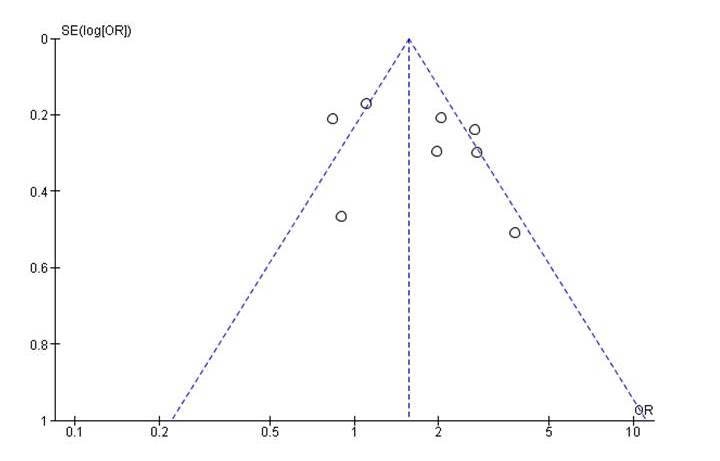
Supplementary Figure 1.** Funnel plot of included studies for the primary outcome (mRS 0-2) showing symmetry of studies suggestive of lack of publication bias.

**Table S2: All 299 studies showing reasons for exclusion**

| **Study Number** | **Included** | **Excluded by Title** | **Excluded by Abstract** | **Excluded by Full Text** | **Reason for excluding full text** | | **Title of paper** |
| --- | --- | --- | --- | --- | --- | --- | --- |
| 1 |  |  | 1 |  | Not the primary publication | | Differential Effect of Baseline Computed Tomographic Angiography Collaterals  on Clinical Outcome in Patients Enrolled in the Interventional Management of  Stroke III Trial. |
| 2 | 1 |  |  |  |  | | Randomized assessment of rapid endovascular treatment of ischemic stroke. |
| 3 | 1 |  |  |  |  | | Endovascular therapy for ischemic stroke with perfusion-imaging selection |
| 4 |  | 1 |  |  | Not AIS | | Embolisation for pulmonary arteriovenous malformation. |
| 5 | 1 |  |  |  |  | | A randomized trial of intraarterial treatment for acute ischemic stroke |
| 6 |  |  | 1 |  | Not an RCT | | Advances in Endovascular Treatment of Acute Ischemic Stroke |
| 7 |  | 1 |  |  | Not an RCT | | A meta-analysis of prospective randomized controlled trials evaluating  endovascular therapies for acute ischemic stroke |
| 8 |  |  | 1 |  | Not a thrombectomy paper | | Surgeon's 30-day outcomes supporting the carotid revascularization  endarterectomy versus stenting trial |
| 9 |  | 1 |  |  | Not an RCT | | Recanalization of acute basilar artery occlusion improves outcomes: a  meta-analysis. |
| 10 |  | 1 |  |  | Not an RCT | | Early reperfusion and clinical outcomes in patients with M2 occlusion:  pooled analysis of the PROACT II, IMS, and IMS II studies |
| 11 |  | 1 |  |  | Not AIS | | Complication rates and center enrollment volume in the carotid  revascularization endarterectomy versus stenting trial |
| 12 |  | 1 |  |  | Not an RCT | | Time is Penumbra: imaging, selection and outcome. The Johann jacob wepfer award |
| 13 |  | 1 |  |  | Not AIS | | The impact of frequency of patient self-testing of prothrombin time on time in  target range within VA Cooperative Study #481: The Home INR Study (THINRS),  a randomized, controlled trial |
| 14 |  |  | 1 |  | Not an RCT | | Adaptive choice of patient subgroup for comparing two treatments |
| 15 |  |  | 1 |  | Not an RCT | | Future directions for intra-arterial therapy for acute ischaemic stroke: is there life  after three negative randomized controlled studies |
| 16 |  |  | 1 |  | Not an RCT (protocol only) | | MR CLEAN, a multicenter randomized clinical trial of endovascular treatment for acute  ischemic stroke in the Netherlands: study protocol for a randomized controlled trial |
| 17 |  | 1 |  |  | Not AIS, not an RCT | | New frontiers in aortic therapy: focus on deep hypothermic circulatory arrest |
| 18 |  | 1 |  |  | Not AIS | | Endovascular treatment for ruptured abdominal aortic aneurysm |
| 19 |  | 1 |  |  | Not AIS | | Percutaneous mitral valve repair for mitral regurgitation in high-risk patients:  results of the EVEREST II study |
| 20 |  |  | 1 |  | Not the primary publication | | Recanalization and clinical outcome of occlusion sites at baseline CT angiography  in the Interventional Management of Stroke III trial |
| 21 |  | 1 |  |  | Not a thrombectomy paper | | The Norwegian tenecteplase stroke trial (NOR-TEST): randomised controlled trial  of tenecteplase vs. alteplase in acute ischaemic stroke |
| 22 |  |  | 1 |  | Not the primary publication | | Drivers of costs associated with reperfusion therapy in acute stroke: the Interventional  Management of Stroke III Trial |
| 23 |  |  | 1 |  | Not the primary publication | | Impact of collaterals on successful revascularization in Solitaire FR with the i  ntention for thrombectomy |
| 24 |  | 1 |  |  | Not an RCT | | State of endovascular therapy for acute ischaemic stroke |
| 25 |  |  | 1 |  | Not the primary publication | | The THRIVE score strongly predicts outcomes in patients treated with the Solitaire  device in the SWIFT and STAR trials |
| 26 |  | 1 |  |  | Not AIS | | Flow diversion versus traditional endovascular coiling therapy: design of the  prospective LARGE aneurysm randomized trial |
| 27 |  | 1 |  |  | Not a thrombectomy paper | | EuroHYP-1: European multicenter, randomized, phase III clinical trial of therapeutic  hypothermia plus best medical treatment vs. best medical treatment alone for acute  ischemic stroke |
| 28 |  |  | 1 |  | Not the primary publication | | Evaluation of interval times from onset to reperfusion in patients undergoing  endovascular therapy in the Interventional Management of Stroke III trial |
| 29 |  | 1 |  |  | Not AIS | | Ultrasound surveillance after CAS and CEA: what's the evidence |
| 30 |  | 1 |  |  | Not an RCT | | In search of the optimized stroke trial design |
| 31 |  |  | 1 |  | Not the primary publication | | Challenges of decision making regarding futility in a randomized trial: the  Interventional Management of Stroke III experience |
| 32 |  | 1 |  |  | Not AIS | | Variability in outcome after elective cerebral aneurysm repair in high-volume  academic medical centers |
| 33 |  |  | 1 |  | Not an RCT | | Thrombolysis and reperfusion: advanced understanding of early management s  trategies in acute ischemic stroke |
| 34 |  | 1 |  |  | Not an RCT | | Collateral lessons from recent acute ischemic stroke trials |
| 35 |  |  | 1 |  | Not an RCT | | Endovascular treatment of acute ischemic stroke: Honolulu shock and thereafter |
| 36 |  | 1 |  |  | Not AIS | | Evolution of transcatheter aortic valve replacement |
| 37 |  | 1 |  |  | Not an RCT | | Society for Neuroscience in Anesthesiology and Critical Care Expert consensus  statement: anesthetic management of endovascular treatment for acute ischemic  stroke*: endorsed by the Society of NeuroInterventional Surgery and the Neurocritical  Care Society |
| 38 |  | 1 |  |  | Not an RCT | | Intracranial stenosis: impact of randomized trials on treatment preferences of  US neurologists and neurointerventionists |
| 39 |  |  | 1 |  | Not an RCT | | Endovascular therapies in acute ischemic stroke |
| 40 |  | 1 |  |  | Not AIS | | When not to implant the multilayer flow modulator: lessons learned from  application outside the indications for use in patients with thoracoabdominal  pathologies. |
| 41 |  |  | 1 |  | Comparing two EVT devices | | Comparison of neurologic and radiographic outcomes with Solitaire versus  Merci/Penumbra systems for acute stroke intervention |
| 42 |  | 1 |  |  | Not AIS | | Endovascular repair of abdominal aortic aneurysm |
| 43 |  | 1 |  |  | Not AIS | | Targeted temperature management after intracerebral hemorrhage (TTM-ICH):  methodology of a prospective randomized clinical trial |
| 44 |  | 1 |  |  | Not an RCT | | Endovascular therapy did not improve expected prognosis after i.v. fibrinolysis:  a retrospective analysis based on a nationwide Danish registry |
| 45 |  |  | 1 |  | Not an RCT | | Endovascular treatment of acute ischemic stroke: the end or just the beginning? |
| 46 |  |  | 1 |  | Not the primary publication | | Alberta Stroke Program early computed tomography score to select patients for  endovascular treatment: Interventional Management of Stroke (IMS)-III Trial |
| 47 |  | 1 |  |  | Not AIS | | Medical management with or without interventional therapy for unruptured brain  arteriovenous malformations (ARUBA): a multicentre, non-blinded, randomised trial |
| 48 |  | 1 |  |  | Not an RCT | | Clinical factors are significant predictors of outcome post intra-arterial therapy for  acute ischaemic stroke: a review |
| 49 |  |  | 1 |  | Not an RCT (protocol only) | | A multicenter, randomized, controlled study to investigate EXtending the time for  Thrombolysis in Emergency Neurological Deficits with Intra-Arterial therapy (  EXTEND-IA). |
| 50 |  | 1 |  |  | Not a thrombectomy paper | | Endovascular therapeutic hypothermia for acute ischemic stroke: ICTuS 2/3 protocol |
| 51 |  | 1 |  |  | Not an RCT | | Efficacy and safety of endovascular treatment versus intravenous thrombolysis for  acute ischemic stroke: a meta-analysis of randomized controlled trials |
| 52 |  | 1 |  |  | Not AIS | | Improved functional status and quality of life in prohibitive surgical risk patients  with degenerative mitral regurgitation after transcatheter mitral valve repair |
| 53 |  | 1 |  |  | Not an RCT | | Endovascular therapy for acute ischemic stroke: a systematic review and meta-analysis |
| 54 |  |  | 1 |  | Not the primary publication | | THRIVE score predicts outcomes with a third-generation endovascular stroke  treatment device in the TREVO-2 trial |
| 55 |  | 1 |  |  | Not an RCT | | Carotid wars episode VI: Return of the standard |
| 56 |  |  | 1 |  | Not the primary publication | | Complications of endovascular treatment for acute stroke in the SWIFT trial with  solitaire and Merci devices |
| 57 |  | 1 |  |  | Not an RCT | | Statement of ESMINT and ESNR regarding recent trials evaluating the endovascular  treatment at the acute stage of ischemic stroke |
| 58 |  |  | 1 |  | Not an RCT | | Advances and challenges in treatment and prevention of ischemic stroke |
| 59 |  | 1 |  |  | Not AIS | | Length of carotid stenosis predicts peri-procedural stroke or death and restenosis  in patients randomized to endovascular treatment or endarterectomy |
| 60 |  |  | 1 |  | Not an RCT | | Carotid artery occlusive disease |
| 61 |  | 1 |  |  | Not AIS | | Long-term results after transcatheter aortic valve implantation: positive and side  effects |
| 62 |  | 1 |  |  | Not an RCT | | The Feinberg Award Lecture 2013: treatment of intracranial atherosclerosis: learning  from the past and planning for the future |
| 63 |  | 1 |  |  | Not AIS | | Medical management of critical limb ischaemia: where do we stand today? |
| 64 |  | 1 |  |  | Not AIS | | Prospective, randomized, open-label phase II trial on concomitant intraventricular  fibrinolysis and low-frequency rotation after severe subarachnoid hemorrhage |
| 65 |  | 1 |  |  | Not AIS | | Surgical trials in intracerebral hemorrhage |
| 66 |  | 1 |  |  | Not an RCT | | The evolution of technology |
| 67 |  | 1 |  |  | Not the primary publication | | Reperfusion therapy for acute ischemic stroke: how should we react to the Third I  nterventional Management of Stroke (IMS III) trial |
| 68 |  | 1 |  |  | Not the primary publication | | What the SWIFT and TREVO II trials tell us about the role of endovascular therapy  for acute stroke |
| 69 |  | 1 |  |  | Not AIS | | Endovascular treatment of intracranial aneurysms in elderly patients: a systematic r  eview and meta-analysis |
| 70 |  |  |  | 1 | Not an RCT | | Endovascular therapy in acute ischemic stroke |
| 71 |  | 1 |  |  | Not AIS | | Predictors of mortality and outcomes of therapy in low-flow severe aortic stenosis:  a Placement of Aortic Transcatheter Valves (PARTNER) trial analysis |
| 72 |  | 1 |  |  | Not an RCT | | Acute stroke trials: the elephant in the room |
| 73 |  | 1 |  |  | Not an RCT | | The role of sonolysis and sonothrombolysis in acute ischemic stroke: a systematic  review and meta-analysis of randomized controlled trials and case-control studies |
| 74 |  |  | 1 |  | Not an RCT | | Intra-arterial reperfusion strategies in acute ischemic stroke |
| 75 |  | 1 |  |  | Not AIS | | The role of carotid stenting for the treatment of carotid disease |
| 76 |  | 1 |  |  | Article in German | | [Acute treatment of cerebral infarction]. |
| 77 |  | 1 |  |  | Article in Spanish | | [Time is still brain: the ball is in the court of neurovascular interventionist treatment]. |
| 78 |  | 1 |  |  | Not AIS | | Comparison of outcomes of patients with ST-segment elevation myocardial  infarction with versus without previous coronary artery bypass grafting (from  the Harmonizing Outcomes With Revascularization and Stents in Acute Myocardial  Infarction [HORIZONS-AMI] trial). |
| 79 |  |  | 1 |  | Not AIS | | The effect of postoperative stroke and myocardial infarction on long-term survival  after carotid revascularization |
| 80 | 1 |  |  |  |  | | A trial of imaging selection and endovascular treatment for ischemic stroke |
| 81 | 1 |  |  |  |  | | Endovascular therapy after intravenous t-PA versus t-PA alone for stroke |
| 82 | 1 |  |  |  |  | | Endovascular treatment for acute ischemic stroke |
| 83 |  |  | 1 |  | Not an RCT | | Advanced imaging to extend the therapeutic time window of acute ischemic stroke |
| 84 |  |  |  | 1 | Not an RCT | | Mechanical clot retrieval in the treatment of acute ischemic stroke |
| 85 |  | 1 |  |  | Not AIS | | Outcomes of endovascular coiling versus surgical clipping in the treatment of  ruptured intracranial aneurysms |
| 86 |  | 1 |  |  | Not a thrombectomy paper | | Feasibility of endovascular and surface cooling strategies in acute stroke |
| 87 |  | 1 |  |  | Not AIS | | Design of the Vitesse Intracranial Stent Study for Ischemic Therapy (VISSIT) trial  in symptomatic intracranial stenosis |
| 88 |  | 1 |  |  | Not AIS | | Clipping versus coiling for ruptured intracranial aneurysms: a systematic review  and meta-analysis |
| 89 |  | 1 |  |  | Not a thrombectomy paper | | Is intra-arterial thrombolysis beneficial for M2 occlusions? Subgroup analysis of  the PROACT-II trial |
| 90 |  | 1 |  |  | Not AIS | | Dual antiplatelet therapy in peripheral arterial disease and after peripheral  percutaneous revascularization |
| 91 |  | 1 |  |  | Not an RCT | | Acute stroke trials and consent |
| 92 |  | 1 |  |  | Not a thrombectomy paper | | Heart-brain signaling in patent foramen ovale-related stroke: differential  plasma proteomic expression patterns revealed with a 2-pass liquid chromatography  -tandem mass spectrometry discovery workflow |
| 93 |  |  | 1 |  | Not an RCT (protocol only) | | SOLITAIRE™ with the intention for thrombectomy (SWIFT) trial: design of a  randomized, controlled, multicenter study comparing the SOLITAIRE™ Flow  Restoration device and the MERCI Retriever in acute ischaemic stroke |
| 94 |  |  | 1 |  | Not AIS | | Randomized controlled trial of symptomatic middle cerebral artery stenosis:  endovascular versus medical therapy in a Chinese population |
| 95 |  | 1 |  |  | Not a thrombectomy paper | | Sonothrombolysis for acute ischaemic stroke |
| 96 |  | 1 |  |  | Not a thrombectomy paper | | Addition of either pioglitazone or a sulfonylurea in type 2 diabetic patients  inadequately controlled with metformin alone: impact on cardiovascular events.  A randomized controlled trial. |
| 97 |  | 1 |  |  | Not a thrombectomy paper | | Safety and efficacy of NA-1 in patients with iatrogenic stroke after endovascular  aneurysm repair (ENACT): a phase 2, randomised, double-blind, placebo-controlled trial. |
| 98 |  | 1 |  |  | Not AIS | | Adjunctive ultrasonography to minimize iodinated contrast administration during  carotid artery stenting: a randomized trial |
| 99 |  | 1 |  |  | Not a thrombectomy paper | | Cerebral embolization in asymptomatic versus symptomatic patients after  carotid stenting. |
| 100 |  | 1 |  |  | Not an RCT | | Endovascular acute ischemic stroke therapy: Society of Vascular and Interventional  Neurology roundtable proceedings. |
| 101 |  |  | 1 |  | Not an RCT | | Retrievable stents, "stentrievers," for endovascular acute ischemic stroke therapy. |
| 102 |  |  | 1 |  | Not an RCT | | Treatment of acute ischemic stroke: systemic or local? |
| 103 |  |  | 1 |  | Not an RCT (protocol only) | | Design and rationale of the Mechanical Retrieval and Recanalization of Stroke Clots  Using Embolectomy (MR RESCUE) Trial |
| 104 |  | 1 |  |  | Not AIS | | Percutaneous transluminal balloon angioplasty and stenting for carotid artery stenosis. |
| 105 |  | 1 |  |  | Not an RCT | | MRI profile and response to endovascular reperfusion after stroke (DEFUSE 2):  a prospective cohort study. |
| 106 |  |  | 1 |  | Not an RCT | | Thrombolytic therapy for acute ischaemic stroke: what can we do to improve  outcomes? |
| 107 |  |  | 1 |  | Comparing two EVT devices | | Solitaire flow restoration device versus the Merci Retriever in patients with acute  ischaemic stroke (SWIFT): a randomised, parallel-group, non-inferiority trial |
| 108 |  |  | 1 |  | Comparing two EVT devices | | Trevo versus Merci retrievers for thrombectomy revascularisation of large vessel  occlusions in acute ischaemic stroke (TREVO 2): a randomised trial. |
| 109 |  | 1 |  |  | Article in German | | [Endovascular treatment for carotid artery stenosis] |
| 110 |  | 1 |  |  | Not a thrombectomy paper | | Intravenous recombinant tissue-type plasminogen activator in the extended time  window and the US Food and Drug Administration: confused about the time |
| 111 |  | 1 |  |  | Not AIS | | Embolisation for pulmonary arteriovenous malformation. |
| 112 |  | 1 |  |  | Not an RCT | | Antiplatelet and anticoagulant drugs for prevention of restenosis/reocclusion  following peripheral endovascular treatment |
| 113 |  |  | 1 |  | Not an RCT | | Endovascular therapy yields significantly superior outcomes for large vessel  occlusions compared with intravenous thrombolysis: is it time to randomize |
| 114 |  | 1 |  |  | Not an RCT | | Stroke outcome measures: a tale of two trials. |
| 115 |  | 1 |  |  | Not AIS | | Cerecyte coil trial: angiographic outcomes of a prospective randomized trial  comparing endovascular coiling of cerebral aneurysms with either cerecyte  or bare platinum coils. |
| 116 |  | 1 |  |  | Not a thrombectomy paper | | Intervention for intracranial atherosclerosis after SAMMPRIS. |
| 117 |  | 1 |  |  | Not an RCT | | Systematic review of outcome after ischemic stroke due to anterior circulation  occlusion treated with intravenous, intra-arterial, or combined  intravenous+intra-arterial thrombolysis. |
| 118 |  | 1 |  |  | Not AIS | | Current status of transcatheter aortic valve replacement. |
| 119 |  | 1 |  |  | Not AIS | | Timing of aneurysm treatment after subarachnoid hemorrhage: relationship  with delayed cerebral ischemia and poor outcome |
| 120 |  | 1 |  |  | Not a thrombectomy paper | | Sonothrombolysis for acute ischaemic stroke |
| 121 |  | 1 |  |  | Not AIS | | What is the evidence for IABP in STEMI with and without cardiogenic shock? |
| 122 |  | 1 |  |  | Not AIS | | Endovascular treatment of carotid artery stenosis: evidences from randomized  controlled trials and actual indications |
| 123 |  | 1 |  |  | Not a thrombectomy paper | | Determinants of effective cooling during endovascular hypothermia |
| 124 |  |  | 1 |  | Not an RCT (protocol only) | | Remote ischemic perconditioning in thrombolysed stroke patients:  randomized study of activating endogenous neuroprotection - design and  MRI measurements |
| 125 |  | 1 |  |  | Not an RCT | | Acute stroke therapy: are we ready for primetime clinical trials |
| 126 |  | 1 |  |  | Not AIS | | Randomized trial of clazosentan in patients with aneurysmal subarachnoid  hemorrhage undergoing endovascular coiling |
| 127 |  | 1 |  |  | Not AIS | | Thrombolysis or anticoagulation for cerebral venous thrombosis: rationale  and design of the TO-ACT trial |
| 128 |  | 1 |  |  | Not AIS | | Cooling in intracerebral hemorrhage (CINCH) trial: protocol of a randomized  German-Austrian clinical trial |
| 129 |  | 1 |  |  | Not AIS | | Treatment of intracranial atherosclerotic disease with a balloon-expandable  paclitaxel eluting stent: procedural safety, efficacy and mid-term patency |
| 130 |  | 1 |  |  | Not AIS | | Intracranial angioplasty and stent placement after stenting and aggressive  medical management for preventing recurrent stroke in intracranial stenosis  (SAMMPRIS) trial: present state and future considerations |
| 131 |  | 1 |  |  | Not AIS | | Carotid endarterectomy and treatment options for carotid occlusive disease |
| 132 |  | 1 |  |  | Not AIS | | Percutaneous transluminal angioplasty and stenting in patients with proximal  vertebral artery stenosis |
| 133 |  | 1 |  |  | Not AIS | | Influence and critique of the INSTEAD Trial (TEVAR versus medical treatment  for uncomplicated type B aortic dissection). |
| 134 |  |  | 1 |  | Not an RCT | | Endovascular treatment strategies for acute ischemic stroke |
| 135 |  | 1 |  |  | Not AIS | | Role of ST2 in non-ST-elevation acute coronary syndrome in the MERLIN-TIMI 36 trial |
| 136 |  | 1 |  |  | Not AIS | | Antiplatelet agents for intermittent claudication |
| 137 |  | 1 |  |  | Not AIS | | A review of the main trials and registries: what we think we do and do not know  about carotid artery stenting |
| 138 |  |  | 1 |  | Not an RCT | | Thrombolysis in ischemic stroke: focus on new treatment strategies |
| 139 |  | 1 |  |  | Not an RCT | | Meta-analysis of randomized intra-arterial thrombolytic trials for the treatment  of acute stroke due to middle cerebral artery occlusion |
| 140 |  | 1 |  |  | Not an RCT | | Endovascular therapy for acute basilar artery occlusion: a review of the literature |
| 141 |  |  | 1 |  | Not a thrombectomy paper | | Administration of edaravone, a free radical scavenger, during t-PA infusion  can enhance early recanalization in acute stroke patients--a preliminary study |
| 142 |  | 1 |  |  | Not AIS | | Interpretation and implications of the prematurely terminated Stenting and  Aggressive Medical Management for Preventing Recurrent Stroke in the  Intracranial Stenosis (SAMMPRIS) trial |
| 143 |  | 1 |  |  | Not an RCT | | Stroke Treatment Academic Industry Roundtable (STAIR) recommendations  for maximizing the use of intravenous thrombolytics and expanding treatment  options with intra-arterial and neuroprotective therapies |
| 144 |  | 1 |  |  | Not AIS | | Occurrence and impact of delayed cerebral ischemia after coiling and after  clipping in the International Subarachnoid Aneurysm Trial (ISAT). |
| 145 |  |  | 1 |  | Not an RCT | | Advances in revascularization for acute ischemic stroke treatment: an update |
| 146 |  |  | 1 |  | Not an RCT | | The Risk of Paradoxical Embolism (RoPE) Study: developing risk models  for application to ongoing randomized trials of percutaneous patent foramen  ovale closure for cryptogenic stroke |
| 147 |  | 1 |  |  | Not AIS | | Randomized clinical trial of open-cell vs closed-cell stents for carotid stenting  and effects of stent design on cerebral embolization |
| 148 |  | 1 |  |  | Not an RCT | | What's new in stroke? The top 10 studies of 2009-2011: part |
| 149 |  | 1 |  |  | Not AIS | | Intracranial stenting with Wingspan: still awaiting a safe landing |
| 150 |  | 1 | 1 |  | Not AIS | | Controversies around carotid stenting |
| 151 |  | 1 |  |  | Not AIS | | The relationship between attenuated plaque identified by intravascular ultrasound  and no-reflow after stenting in acute myocardial infarction: the HORIZONS-AMI  (Harmonizing Outcomes With Revascularization and Stents in Acute Myocardial  Infarction) trial. |
| 152 |  |  |  | 1 | Not an RCT (protocol only) | | SYNTHESIS expansion: design of a nonprofit, pragmatic, randomized, controlled  trial on the best fast-track endovascular treatment vs. standard intravenous  alteplase for acute ischemic stroke. |
| 153 |  | 1 |  |  | Not AIS | | Incidence of cardiovascular events and death after open or endovascular repair of  abdominal aortic aneurysm in the randomized EVAR trial. |
| 154 |  |  | 1 |  | Not an RCT | | The endovascular management of ischemic stroke |
| 155 |  | 1 |  |  | Not AIS | | Preventive strategies of renal insufficiency in patients with diabetes undergoing  intervention or arteriography (the PREVENT Trial). |
| 156 |  | 1 |  |  | Not AIS | | Endovascular stenting or carotid endarterectomy for treatment of carotid  stenosis: a meta-analysis. |
| 157 |  | 1 |  |  | Not AIS | | Endovascular cooling and endothelial activation in hemorrhagic stroke patients |
| 158 |  |  | 1 |  | Not a thrombectomy paper | | Randomized clinical trial comparing percutaneous closure of patent foramen ovale  (PFO) using the Amplatzer PFO Occluder with medical treatment in patients with  cryptogenic embolism (PC-Trial): rationale and design. |
| 159 |  | 1 |  |  | Not AIS | | Therapeutic hypothermia for out-of-hospital cardiac arrest: an update for  neurosurgeons. |
| 160 |  |  | 1 |  | Not AIS | | 2011 ASA/ACCF/AHA/AANN/AANS/ACR/ASNR/CNS/SAIP/ SCAI/SIR/SNIS/SVM/SVS guideline on  the management of patients with extracranial carotid and vertebral artery  disease: executive summary: a report of the American College of Cardiology  Foundation/American Heart Association Task Force on Practice Guidelines, and the  American Stroke Association, American Association of Neuroscience Nurses,  American Association of Neurological Surgeons, American College of Radiology,  American Society of Neuroradiology, Congress of Neurological Surgeons, Society of  Atherosclerosis Imaging and Prevention, Society for Cardiovascular Angiography  and Interventions, Society of Interventional Radiology, Society of  NeuroInterventional Surgery, Society for Vascular Medicine, and Society for  Vascular Surgery. |
| 161 |  |  | 1 |  | Not AIS | | 2011 ASA/ACCF/AHA/AANN/AANS/ACR/ASNR/CNS/SAIP/ SCAI/SIR/SNIS/SVM/SVS guideline on  the management of patients with extracranial carotid and vertebral artery  disease: executive summary: a report of the American College of Cardiology  Foundation/American Heart Association Task Force on Practice Guidelines, and the  American Stroke Association, American Association of Neuroscience Nurses,  American Association of Neurological Surgeons, American College of Radiology,  American Society of Neuroradiology, Congress of Neurological Surgeons, Society of  Atherosclerosis Imaging and Prevention, Society for Cardiovascular Angiography  and Interventions, Society of Interventional Radiology, Society of  NeuroInterventional Surgery, Society for Vascular Medicine, and Society for  Vascular Surgery. |
| 162 |  |  | 1 |  | Not AIS | | [Recent advances in the treatment of superficial vein thrombosis and extracranial  carotid artery stenosis]. |
| 163 |  |  | 1 |  | Not an RCT | | Does intra-arterial thrombolysis have a role as first-line intervention in acute  ischaemic stroke? |
| 164 |  | 1 |  |  | Not AIS | | Strategies for subacute/chronic type B aortic dissection: the Investigation Of  Stent Grafts in Patients with type B Aortic Dissection (INSTEAD) trial 1-year  outcome. |
| 165 |  |  | 1 |  | Not a thrombectomy paper | | Influence of prophylactic, endovascularly based normothermia on inflammation in  patients with severe cerebrovascular disease: a prospective, randomized trial. |
| 166 |  |  | 1 |  | Not an RCT | | [Actual review of diagnostics and endovascular therapy of intracranial arterial  stenoses]. |
| 167 |  | 1 |  |  | Not AIS | | Do we need a new carotid artery stenting trial? |
| 168 |  |  |  | 1 | Not the primary publication | | Effects of sex on mechanical embolectomy outcome. |
| 169 |  | 1 |  |  | Not AIS | | Preventing vasospasm improves outcome after aneurysmal subarachnoid hemorrhage:  rationale and design of CONSCIOUS-2 and CONSCIOUS-3 trials. |
| 170 |  |  | 1 |  | Not AIS | | Endovascular treatment or neurosurgical clipping of ruptured intracranial  aneurysms: effect on angiographic vasospasm, delayed ischemic neurological  deficit, cerebral infarction, and clinical outcome. |
| 171 |  |  | 1 |  | Not a thrombectomy paper | | Intravenous thrombolysis plus hypothermia for acute treatment of ischemic stroke  (ICTuS-L): final results. |
| 172 |  |  | 1 |  | Not an RCT | | Low patient enrollment sites in multicenter randomized clinical trials of  cerebrovascular diseases: associated factors and impact on trial outcomes. |
| 173 |  | 1 |  |  | Not AIS | | Heparin versus bivalirudin for carotid artery stenting using proximal  endovascular clamping for neuroprotection: results from a prospective randomized  study. |
| 174 |  | 1 |  |  | Not AIS | | Are symptomatic patients currently appropriate candidates for carotid artery  stenting? Yes. |
| 175 |  |  | 1 |  | Not an RCT | | Stroke: a modern history |
| 176 |  | 1 |  |  | Not AIS | | Improved cognitive outcomes with endovascular coiling of ruptured intracranial  aneurysms: neuropsychological outcomes from the International Subarachnoid  Aneurysm Trial (ISAT). |
| 177 |  | 1 |  |  | Not AIS | | Interventions for treating brain arteriovenous malformations in adults. |
| 178 |  | 1 |  |  | Not AIS | | The EVEREST II Trial: design and rationale for a randomized study of the evalve  mitraclip system compared with mitral valve surgery for mitral regurgitation. |
| 179 |  | 1 |  |  | Not AIS | | Impact of systemic inflammatory response syndrome on vasospasm, cerebral  infarction, and outcome after subarachnoid hemorrhage: exploratory analysis of  CONSCIOUS-1 database. |
| 180 |  | 1 |  |  | Not AIS | | Carotid endarterectomy versus carotid angioplasty with or without stenting for  treatment of carotid artery stenosis: an updated meta-analysis of randomized  controlled trials. |
| 181 |  | 1 |  |  | Not AIS | | The Carotid Revascularization Endarterectomy versus Stenting Trial: credentialing  of interventionalists and final results of lead-in phase. |
| 182 |  | 1 |  |  | Not AIS | | Safety and efficacy of thrombectomy in patients undergoing primary percutaneous  coronary intervention for acute ST elevation MI: a meta-analysis of randomized  controlled trials. |
| 183 |  |  |  | 1 | Limited use of throbectomy devices | | Intra-arterial or intravenous thrombolysis for acute ischemic stroke? The SYNTHESIS pilot trial. |
| 184 |  | 1 |  |  | Not AIS | | [Evidences demonstrating the effects of anti-atherosclerotic actions of  pioglitazone--special emphasis on PROactive Study and PERISCOPE Study]. |
| 185 |  |  |  | 1 | Not an RCT (comparing new protocol to previous protocol at the same centre) | | Drip, ship, and retrieve: cooperative recanalization therapy in acute basilar  artery occlusion. |
| 186 |  | 1 |  |  | Not AIS | | Does EVAR alter the rate of cardiovascular events in patients with abdominal  aortic aneurysm considered unfit for open repair? Results from the randomised  EVAR trial 2. |
| 187 |  | 1 |  |  | Not a thrombectomy paper | | Effect of intravenous tirofiban and aspirin in reducing short-term and long-term  neurologic deficit in patients with ischemic stroke: a double-blind randomized  trial. |
| 188 |  |  | 1 |  | Not an RCT | | Randomised trials of endovascular treatment of stroke are needed |
| 189 |  |  | 1 |  | Not an RCT | | Randomised trials of endovascular treatment of stroke are needed |
| 190 |  | 1 |  |  | Not AIS | | Carotid artery stenting: a systematic review of randomized clinical trials |
| 191 |  | 1 |  |  | Not AIS | | Randomized comparison of strategies for type B aortic dissection: the  INvestigation of STEnt Grafts in Aortic Dissection (INSTEAD) trial. |
| 192 |  |  | 1 |  | Not AIS | | The rationale behind "A Randomized Trial of Unruptured Brain AVMs" (ARUBA). |
| 193 |  |  | 1 |  | Not AIS | | Carotis stenosis-open surgery and endovascular treatment |
| 194 |  |  | 1 |  | Not an RCT | | Intracranial atherosclerotic disease |
| 195 |  | 1 |  |  | Not AIS | | Carotid artery stenting vs. endarterectomy |
| 196 |  |  | 1 |  | Not an RCT | | Prospects of thrombolytic therapy for acute ischemic stroke |
| 197 |  |  | 1 |  | Not a thrombectomy paper | | Prophylactic, endovascularly based, long-term normothermia in ICU patients with  severe cerebrovascular disease: bicenter prospective, randomized trial. |
| 198 |  |  | 1 |  | Not AIS | | Long-term risk of carotid restenosis in patients randomly assigned to  endovascular treatment or endarterectomy in the Carotid T Vertebral Artery  Transluminal Angioplasty Study (CAVATAS): long-term follow-up of a randomised  trial. |
| 199 |  | 1 |  |  | Not AIS | | Endovascular treatment with angioplasty or stenting versus endarterectomy in  patients with carotid artery stenosis in the Carotid and Vertebral Artery  Transluminal Angioplasty Study (CAVATAS): long-term follow-up of a randomised  trial. |
| 200 |  | 1 |  |  | Not AIS | | A multimodal concept in patients after severe aneurysmal subarachnoid hemorrhage:  results of a controlled single centre prospective randomized multimodal phase  I/II trial on cerebral vasospasm. |
| 201 |  | 1 |  |  | Not AIS | | Statins and coronary artery bypass graft surgery: preoperative and postoperative  efficacy and safety. |
| 202 |  | 1 |  |  | Not AIS | | Carotid artery stenosis-an evidence-based review of surgical and non-surgical  treatments. |
| 203 |  |  | 1 |  | Not AIS | | The GALA trial: will it influence clinical practice? |
| 204 |  |  | 1 |  | Not an RCT | | Treatment and outcomes of acute basilar artery occlusion in the Basilar Artery  International Cooperation Study (BASICS): a prospective registry study. |
| 205 |  | 1 |  |  | Not AIS | | Long-term outcome of endovascular treatment versus medical care for carotid  artery stenosis in patients not suitable for surgery and randomised in the  Carotid and Vertebral Artery Transluminal Angioplasty study (CAVATAS). |
| 206 |  | 1 |  |  | Not AIS | | Effect of antiplatelet therapy for endovascular coiling in aneurysmal  subarachnoid hemorrhage. |
| 207 |  | 1 |  |  | Not AIS | | [Late results of endovascular coronary revascularization in patients with type 2  diabetes mellitus]. |
| 208 |  | 1 |  |  | Not AIS | | Prasugrel compared with clopidogrel in patients undergoing percutaneous coronary  intervention for ST-elevation myocardial infarction (TRITON-TIMI 38):  double-blind, randomised controlled trial. |
| 209 |  | 1 |  |  | Not AIS | | Randomized controlled trials comparing endarterectomy and endovascular treatment  for carotid artery stenosis: a Cochrane systematic review. |
| 210 |  | 1 |  |  | Not AIS | | Magnesium and aspirin treatment in patients with subarachnoid haemorrhage.  Comparison of effects after endovascular and neurosurgical aneurysm occlusion. |
| 211 |  | 1 |  |  | Not AIS | | Efficacy of cilostazol after endovascular therapy for femoropopliteal artery  disease in patients with intermittent claudication. |
| 212 |  |  | 1 |  | Not an RCT | | Endovascular therapy for acute ischemic stroke |
| 213 |  |  |  | 1 | Not an RCT | | Comparison of mechanical embolectomy and intraarterial thrombolysis in acute  ischemic stroke within the MCA: MERCI and Multi MERCI compared to PROACT II. |
| 214 |  | 1 |  |  | Not AIS | | Transferring patients with ST-segment elevation myocardial infarction for  mechanical reperfusion: a meta-regression analysis of randomized trials. |
| 215 |  | 1 |  |  | Not AIS | | Asymptomatic unruptured intracranial aneurysms: approach to screening and  treatment. |
| 216 |  | 1 |  |  | Not AIS | | High rate of restenosis after carotid artery stenting in patients with high-grade  internal carotid artery stenosis. Medium-term follow-up. |
| 217 |  | 1 |  |  | Not AIS | | International subarachnoid aneurysm trial of neurosurgical clipping versus  endovascular coiling: subgroup analysis of 278 elderly patients. |
| 218 |  | 1 |  |  | Not AIS | | Stent-protected angioplasty versus carotid endarterectomy in patients with  carotid artery stenosis: meta-analysis of randomized trial data. |
| 219 |  | 1 |  |  | Not AIS | | Alert for increased long-term follow-up after carotid artery stenting: results of  a prospective, randomized, single-center trial of carotid artery stenting vs  carotid endarterectomy. |
| 220 |  |  | 1 |  | Not AIS | | Current management of extracranial carotid artery disease |
| 221 |  | 1 |  |  | Not AIS | | Retrojugular versus ventrojugular approach to carotid bifurcation for eversion  endarterectomy: a prospective randomized trial. |
| 222 |  | 1 |  |  | Not AIS | | Endarterectomy or carotid artery stenting: the quest continues |
| 223 |  |  | 1 |  | Not a thrombectomy paper | | PFO and stroke: what are the data? |
| 224 |  | 1 |  |  | Not an RCT | | Regarding "Luebke T, Aleksic M, Brunkwall J. Meta-analysis of randomized trials  comparing carotid endarterectomy and endovascular treatment". Eur J Vasc Endovasc  Surg 2007;34:470-479. |
| 225 |  | 1 |  |  | Not AIS | | Interventional neuroradiology in the treatment of cerebral venous thrombosis |
| 226 |  |  | 1 |  | Not an RCT | | Endovascular treatment of acute ischaemic stroke. - Review |
| 227 |  | 1 |  |  | Not AIS | | Percutaneous transluminal angioplasty and stenting for carotid artery stenosis. |
| 228 |  | 1 |  |  | Not AIS | | Contemporary management of carotid stenosis: carotid endarterectomy is here to  stay. |
| 229 |  | 1 |  |  | Not AIS | | Commentary on "Carotid artery stenting will replace carotid endarterectomy". |
| 230 |  | 1 |  |  | Not AIS | | Meta-analysis of randomized trials comparing carotid endarterectomy and  endovascular treatment. |
| 231 |  | 1 |  |  | Not AIS | | Endovascular or surgical treatment for carotid artery stenosis? |
| 232 |  | 1 |  |  | Not AIS | | Subtle cerebral damage after shunting vs non shunting during carotid  endarterectomy. |
| 233 |  | 1 |  |  | Not AIS | | Duplex scan surveillance after carotid angioplasty and stenting: a rational  definition of stent stenosis. |
| 234 |  |  | 1 |  | Not an RCT | | Beyond intravenous thrombolysis. |
| 235 |  | 1 |  |  | Not AIS | | [Carotid artery stenosis]. |
| 236 |  | 1 |  |  | Not AIS | | Carotid endarterectomy versus carotid stenting: an updated review of randomized  trials and subgroup analyses. |
| 237 |  | 1 |  |  | Not AIS | | Retreatment of ruptured cerebral aneurysms in patients randomized by coiling or  clipping in the International Subarachnoid Aneurysm Trial (ISAT). |
| 238 |  | 1 |  |  | Not AIS | | Long-term outcome after angioplasty and stenting for symptomatic vertebral artery  stenosis compared with medical treatment in the Carotid And Vertebral Artery  Transluminal Angioplasty Study (CAVATAS): a randomized trial. |
| 239 |  |  | 1 |  | Not an RCT | | Experimental treatments for acute ischaemic stroke. |
| 240 |  | 1 |  |  | Not AIS | | [Coronary syndromes in the elderly]. |
| 241 |  | 1 |  |  | Not AIS | | SPACE and EVA-3S trials: the need of standards for carotid stenting. |
| 242 |  | 1 |  |  | Not AIS | | Carotid artery stenting versus carotid endarterectomy: current status. |
| 243 |  | 1 |  |  | Not AIS | | [Interventional treatment of extracranial carotid stenoses: current status,  requirements and indications]. |
| 244 |  |  | 1 |  | Not a thrombectomy paper | | A trial of therapeutic hypothermia via endovascular approach in awake patients  with acute ischemic stroke: methodology. |
| 245 |  | 1 |  |  | Not AIS | | Endovascular coiling versus neurosurgical clipping for patients with aneurysmal  subarachnoid haemorrhage. |
| 246 |  | 1 |  |  | Not AIS | | Secondary prevention of macrovascular events in patients with type 2 diabetes in  the PROactive Study (PROspective pioglitAzone Clinical Trial In macroVascular  Events): a randomised controlled trial. |
| 247 |  | 1 |  |  | Not AIS | | Carotid Revascularization Using Endarterectomy or Stenting Systems (CaRESS) phase  I clinical trial: 1-year results. |
| 248 |  | 1 |  |  | Not AIS | | The impact of patient self-testing of prothrombin time for managing  anticoagulation: rationale and design of VA Cooperative Study #481--the Home INR  Study (THINRS). |
| 249 |  | 1 |  |  | Not AIS | | [Possible treatments for patients with symptomatic carotid artery stenosis]. |
| 250 |  | 1 |  |  | Not AIS | | Percutaneous transluminal angioplasty and stenting for vertebral artery stenosis. |
| 251 |  | 1 |  |  | Not an RCT | | Alteplase and ischaemic stroke: have new reviews of old data helped? |
| 252 |  | 1 |  |  | Not AIS | | Safety and efficacy of endovascular treatment of carotid artery stenosis compared  with carotid endarterectomy: a Cochrane systematic review of the randomized  evidence. |
| 253 |  | 1 |  |  | Not AIS | | Antiplatelet and anticoagulant drugs for prevention of restenosis/reocclusion  following peripheral endovascular treatment. |
| 254 |  | 1 |  |  | Not AIS | | Restenosis after carotid angioplasty, stenting, or endarterectomy in the Carotid  and Vertebral Artery Transluminal Angioplasty Study (CAVATAS). |
| 255 |  | 1 |  |  | Not an RCT | | [Consensus and controversy in the treatment of ischemic cerebrovascular  diseases]. |
| 256 |  | 1 |  |  | Not an RCT | | [Vascular surgery in the elderly]. |
| 257 |  | 1 |  |  | Not AIS | | Patient selection for revascularization in cervical carotid artery disease:  angioplasty and stenting vs. endarterectomy. |
| 258 |  | 1 |  |  | Not AIS | | Carotid endarterectomy in patients with contralateral carotid occlusion. |
| 259 |  | 1 |  |  | Not AIS | | Carotid endarterectomy: who is the high-risk patient? |
| 260 |  | 1 |  |  | Not AIS | | Combined Abciximab REteplase Stent Study in acute myocardial infarction (CARESS  in AMI). |
| 261 |  | 1 |  |  | Not AIS | | Long-term cardiovascular morbidity, mortality, and reintervention after  endovascular treatment in patients with iliac artery disease: The Dutch Iliac  Stent Trial Study. |
| 262 |  |  | 1 |  | Not a thrombectomy paper | | Cooling for Acute Ischemic Brain Damage (COOL AID): a feasibility trial of  endovascular cooling. |
| 263 |  | 1 |  |  | Not AIS | | Comparison of endovascular and surface cooling during unruptured cerebral  aneurysm repair. |
| 264 |  | 1 |  |  | Not AIS | | [Controversies in the treatment of carotid stenoses. Present state of research  and evidence-based medicine]. |
| 265 |  | 1 |  |  | Not AIS | | International carotid stenting study: protocol for a randomised clinical trial  comparing carotid stenting with endarterectomy in symptomatic carotid artery  stenosis. |
| 266 |  | 1 |  |  | Not AIS | | Carotid artery stenting:the need for randomised trials. |
| 267 |  | 1 |  |  | Not AIS | | Impact of intravenous beta-blockade before primary angioplasty on survival in  patients undergoing mechanical reperfusion therapy for acute myocardial  infarction. |
| 268 |  | 1 |  |  | Not AIS | | Percutaneous transluminal angioplasty and stenting for carotid artery stenosis. |
| 269 |  | 1 |  |  | Not AIS | | Carotid endarterectomy: a review. |
| 270 |  | 1 |  |  | Not AIS | | Infusion techniques for peripheral arterial thrombolysis. |
| 271 |  | 1 |  |  | Not AIS | | Effect of treatment of carotid artery stenosis on blood pressure: a comparison of  hemodynamic disturbances after carotid endarterectomy and endovascular treatment. |
| 272 |  | 1 |  |  | Not AIS | | Extracranial carotid artery stenosis. |
| 273 |  | 1 |  |  | Not AIS | | Carotid artery stenting: current status and future directions. |
| 274 |  | 1 |  |  | Not AIS | | Neurosurgical clipping versus endovascular coiling of patients with ruptured  intracranial aneurysms. |
| 275 |  | 1 |  |  | Not AIS | | Inter-hospital transport for primary angioplasty does not compromise left  ventricular function: six-month echocardiographic follow-up of the PRAGUE 1  Study. |
| 276 |  | 1 |  |  | Not AIS | | [Stents in the treatment of supraaortic vessel stenosis]. |
| 277 |  | 1 |  |  | Not AIS | | [Secondary prevention after ischemic stroke]. |
| 278 |  | 1 |  |  | Not AIS | | Carotid stent angioplasty: the role of cerebral protection devices. |
| 279 |  | 1 |  |  | Not AIS | | S-nitrosoglutathione reduces asymptomatic embolization after carotid angioplasty. |
| 280 |  | 1 |  |  | Not an RCT | | Acute stroke therapy: beyond i.v. tPA. |
| 281 |  | 1 |  |  | Not an RCT | | Innovative Strategies in the Management of Acute Stroke. |
| 282 |  | 1 |  |  | Not AIS | | Surgery versus thrombolysis for acute limb ischaemia: initial management. |
| 283 |  | 1 |  |  | Not AIS | | Magnesium sulfate therapy after aneurysmal subarachnoid hemorrhage. |
| 284 |  |  | 1 |  | Not an RCT | | Innovative strategies in the management of acute stroke. |
| 285 |  | 1 |  |  | Not AIS | | Randomized study comparing cardiac response in endovascular and open abdominal  aortic aneurysm repair. |
| 286 |  | 1 |  |  | Not AIS | | Endovascular versus surgical treatment in patients with carotid stenosis in the  Carotid and Vertebral Artery Transluminal Angioplasty Study (CAVATAS): a  randomised trial. |
| 287 |  | 1 |  |  | Not AIS | | Carotid angioplasty-stent: clinical experience and role for clinical trials. |
| 288 |  | 1 |  |  | Not AIS | | Carotid angioplasty and stenting. |
| 289 |  | 1 |  |  | Not AIS | | Carotid stent placement for extracranial carotid artery disease: current state of  the art. |
| 290 |  | 1 |  |  | Not AIS | | Outcomes of early endovascular versus surgical treatment of ruptured cerebral  aneurysms. A prospective randomized study. |
| 291 |  | 1 |  |  | Not AIS | | [The safety and efficacy of systemic salvage thrombolysis in acute myocardial  infarct]. |
| 292 |  | 1 |  |  | Not AIS | | Efficacy of rescue thrombolysis in patients with acute myocardial infarction:  preliminary findings. |
| 293 |  | 1 |  |  | Not AIS | | Low- versus high-dose recombinant urokinase for the treatment of chronic  saphenous vein graft occlusion |
| 294 |  | 1 |  |  | Not AIS | | Update on mechanical revascularization in acute myocardial infarction: which role  and when? |
| 295 |  | 1 |  |  | Not AIS | | Thrombolysis is superior to heparin for non-obstructive mitral mechanical valve  thrombosis. |
| 296 |  | 1 |  |  | Not AIS | | The role of carotid angioplasty and stenting. |
| 297 |  | 1 |  |  | Not AIS | | Intra-arterially administered papaverine for the treatment of symptomatic  cerebral vasospasm. |
|  | 6 | 212 | 73 | 7 |  | | cerebral vasospasm. |
|  |  |  |  |  |  | |  |
|  | 2 RCTS were too recent to be identified via search and mesh terms at the time of the searches | | | | | | Stent-retriever thrombectomy after intravenous t-PA vs. t-PA alone in stroke |
|  |  |  |  |  | |  | Thrombectomy within 8 hours after symptom onset in ischemic stroke |
|  | 8 |  |  |  | |  |  |

**Table 3: Quality of RCTs using the CASP Randomised Controlled Trials Checklist**

| **Trial, Author, Year** | **Clearly focused issues** | **Random-**  **ization** | **Blinding** | **Similar at start** | **Groups treated equally** | **All patients accounted for at end** | **Size of treatment effect** | **Precision of treatment effect** | **Applied to local context** | **All clinically important outcomes** | **Benefits worth harms and costs** |
| --- | --- | --- | --- | --- | --- | --- | --- | --- | --- | --- | --- |
| **ESCAPE**  Goyal, 2015 | yes | yes | yes | yes | yes | yes | Significant | See table 2 | yes | yes | Yes |
| **EXTEND 1A**  Campbell, 2015 | yes | yes | yes | yes | yes | yes | Significant | See table 2 | yes | yes | Yes |
| **IMS III**  Broderick, 2013 | yes | yes | yes | yes | yes | yes | Not significant | See table 2 | yes | yes | Unclear |
| **MR CLEAN**  Berkhemer, 2015 | yes | yes | yes | yes | yes | yes | Significant | See table 2 | yes | yes | Yes |
| **MR RESCUE**  Kidwell, 2013 | yes | yes | yes | Treatment groups had fewer with congestive heart failure | yes | yes | Not significant | See table 2 | yes | yes | Unclear |
| **REVASCAT**  Jovin, 2015 | yes | yes | yes | yes | yes | yes | Significant | See table 2 | yes | yes | Yes |
| **SWIFT PRIME**  Saver, 2015 | yes | yes | yes | yes | yes | yes | Significant | See table 2 | yes | yes | Yes |
| **SYNTHESIS**  Ciccone, 2013 | yes | yes | yes | Treatment group had fewer with atrial fibrillation | Treatment group was not given t-PA while waiting | yes | Not significant | See table 2 | yes | yes | Unclear |
